# A Pharmacological Dose of Liraglutide Improves Mitochondrial Performance in Mouse Leydig Cells

**DOI:** 10.3390/ijms26188903

**Published:** 2025-09-12

**Authors:** Bruno Oliveira-Lopes, Patrícia C. Braga, Pedro F. Oliveira, Marco G. Alves, Raquel L. Bernardino

**Affiliations:** 1 Unit for Multidisciplinary Research in Biomedicine (UMIB), School of Medicine and Biomedical Sciences (ICBAS), University of Porto, 4050-313 Porto, Portugal; 2 Laboratory for Integrative and Translational Research in Population Health (ITR), 4050-600 Porto, Portugal; 3 LAQV-REQUIMTE and Department of Chemistry, University of Aveiro, 3010-193 Aveiro, Portugal; 4 Institute of Biomedicine (iBiMED), Department of Medical Sciences, University of Aveiro, 3810-193 Aveiro, Portugal

**Keywords:** Leydig cells, liraglutide, male infertility, metabolic disorders, mitochondrial function, steroidogenesis

## Abstract

Male fertility has declined over the years, partly due to metabolic disorders such as obesity and Type 2 diabetes. Antidiabetic drugs, including GLP-1 receptor agonists like liraglutide, are widely used to manage these conditions and aid in weight loss. Within the male reproductive tract, Leydig cells (LCs) are essential since they produce testosterone. Notably, the influence of antidiabetics on LCs remains a subject of limited investigation. Herein, we aimed to evaluate the effect of liraglutide on the physiology of LCs. To this end, we cultured LCs (BLTK1 cell line) without (control) or in the presence of selected concentrations of liraglutide. We then assessed their metabolic viability, cell proliferation, LDH release, ROS production, mitochondrial membrane potential, and in vivo mitochondrial cell performance, as well as the number of mtDNA copies. We also measured androstenedione production. Our results showed that liraglutide at pharmacological and supra-pharmacological concentrations increased the metabolic viability of LCs and reduced ROS production at all concentrations. Furthermore, the pharmacological concentration of liraglutide increased the basal respiration, maximal respiration, proton leak, and oxygen consumption rate related to ATP-linked production. Androstenedione production remained unchanged, which may be related to the inherent limitations of the cell line in supporting steroidogenesis. Overall, our findings suggest that liraglutide exhibits a potential protective effect on LC function, particularly by enhancing metabolic viability, reducing oxidative stress, and improving mitochondrial performance, highlighting its potential beyond the established role in diabetes and weight management.

## 1. Introduction

Metabolic diseases, namely obesity and Diabetes Mellitus (DM), increasingly affect young people of reproductive age. The average age of diagnosis has been decreasing throughout the years, and young men seem to be more and more affected, which has an impact on their fertility [1,2,3,4].

Over the years, numerous drugs have been introduced to the market to manage and mitigate the damage caused by metabolic diseases. Liraglutide is one of the most used glucagon-like peptide 1 receptor agonists (GLP-1RAs), having 97% homology with human GLP-1 [5]. In 2010, it was approved by the Food and Drug Administration (FDA) as an addition to a suitable diet and exercise to ameliorate glycemic control in adults with Type 2 DM (T2DM) [3]. Additionally, liraglutide is administered to obese individuals, with or without T2DM, due to its weight loss benefits [6].

GLP-1 is an incretin hormone that is produced by intestinal cells upon sensing glucose ingestion [7]. When secreted, GLP-1 assumes its active form GLP-1(7-36)NH2, but it lasts only about 2–3 min in the bloodstream since its N-terminal end is cleaved by dipeptidyl dipeptidase-4 (DPP-4) originating GLP-1(9-36)NH2 [8]. Overall, GLP-1 increases glucose-dependent insulin production by pancreatic β-cells, decreases glucagon secretion, slows gastric emptying, and increases satiety by gastric distention [9,10]. GLP-1 also influences the activity of hypothalamic gonadotropin-releasing hormone (GnRH) neurons and testicular development [11]. In addition, GLP-1 indirectly induces *Kiss-1* gene expression, increasing kisspeptin production, which is vital for maintaining fertility [12]. Through the years, there have been numerous reports on the presence of GLP-1 in the male reproductive system. GLP-1 receptor (GLP-1R) was identified in human and mouse testis, specifically in Leydig cells (LCs) and Sertoli cells (SCs) [13,14,15]. In human SCs, GLP-1 has the capacity to modulate the metabolic and oxidative profile, namely glucose consumption, lactate production, and mitochondrial membrane potential [15]. Male GLP-1R knockout mice displayed reduced testis and seminal vesicle weights in addition to deficient glucose metabolism [16].

Some studies show that GLP-1RAs have a beneficial effect on the male reproductive system. Zhang and collaborators have reported that treatment with exenatide (GLP-1RA) improved the quality and motility of sperm in hypogonadal mouse models [17]. Obese patients with hypogonadism treated with liraglutide demonstrated a significant increase in testosterone production [18] and a significant improvement in sperm parameters after 4 months of liraglutide treatment compared to testosterone replacement therapy [19]. Conversely, liraglutide has been reported to reduce sperm concentration and motility in a case report [20]. In another study in diabetic adult mice, liraglutide caused a considerable reduction in sperm count, motility, DNA integrity, and viability [21]. Taking this into account, it is safe to say that GLP-1RAs play a relevant role in the male reproductive system, although with controversial results.

Therefore, it is important to study the role of liraglutide in male reproduction, and in this study, we aim to evaluate the effect of this GLP-1RA on the bioenergetics of LCs.

## 2. Results

### 2.1. Liraglutide Increased Metabolic Viability Without Causing Cytotoxicity in Mouse Leydig Cells

**Figure 1:**
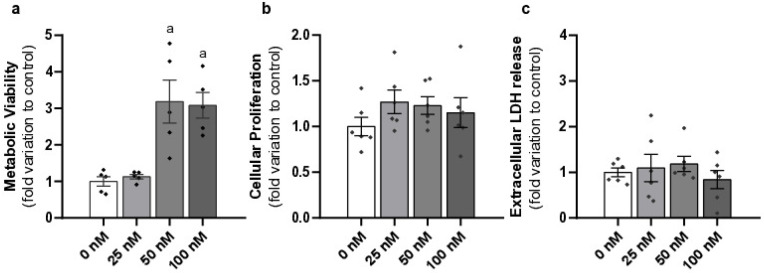
Effects of different concentrations of liraglutide on Leydig cell (LC) metabolic viability (**a**), proliferation (**b**), and extracellular LDH release (**c**). LCs were exposed to sub-pharmacological (25 nM), pharmacological (50 nM), and supra-pharmacological concentrations (100 nM) of liraglutide. Results are expressed as mean ± SEM (*n* = 6 for each condition), in fold variation to the control group (0 nM liraglutide). Significantly different results (*p* < 0.05) are indicated as: a—relative to control (0 nM liraglutide), ^◆^—represent each *n*.

The exposure to pharmacological (50 nM) and supra-pharmacological (100 nM) concentrations of liraglutide increased LCs’ metabolic viability (3.18 ± 0.59 and 3.08 ± 0.34—fold variation to control, respectively, *p* < 0.0001) compared to the control group (1.00 ± 0.13). The cells exposed to the sub-pharmacological concentration of liraglutide (1.13 ± 0.06—fold variation to control) do not differ in metabolic viability compared to those of the control group (Figure 1a).

The impact of liraglutide on LCs’ viability and cytotoxicity was also evaluated. LCs exposed to different concentrations of liraglutide (25 nM–1.27 ± 0.13, 50 nM–1.23 ± 0.10, and 100 nM–1.15 ± 0.16, fold variation to control) do not show changes in cell proliferation relative to the control group (1.00 ± 0.10) (Figure 1b). There were also no differences found in extracellular LDH release in LCs treated with liraglutide (25 nM–1.09 ± 0.30, 50 nM–1.18 ± 0.16, and 100 nM–0.84 ± 0.20—fold variation to control) compared to those of the control group (1.00 ± 0.09) (Figure 1c).

### 2.2. Liraglutide Decreased ROS Production and Did Not Alter the Mitochondrial Membrane Potential of Mouse Leydig Cells

**Figure 2:**
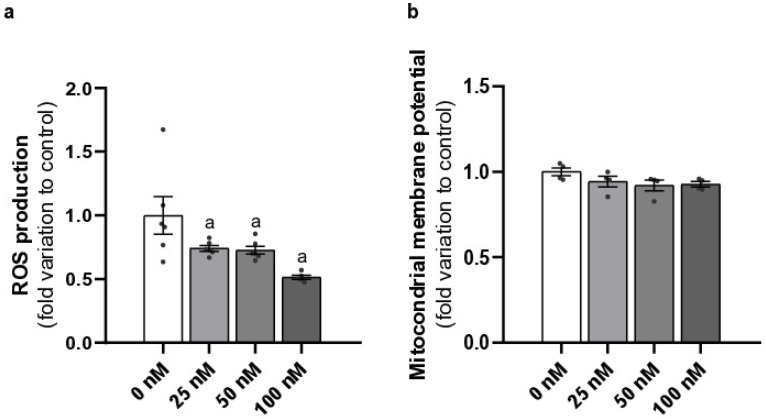
Effects of different concentrations of liraglutide on Leydig cell (LC) ROS production (**a**) and mitochondrial membrane potential (**b**). LCs were exposed to sub-pharmacological (25 nM), pharmacological (50 nM), and supra-pharmacological concentrations (100 nM) of liraglutide. Results are expressed as mean ± SEM (*n* = 6 for each condition), in fold variation to the control group (0 nM liraglutide). Significantly different results (*p* < 0.05) are indicated as: a—relative to control (0 nM liraglutide), ^●^—represent each *n*.

Exposure of LCs to all liraglutide concentrations (25, 50, and 100 nM) decreased ROS production (0.74 ± 0.02, *p* = 0.0319; 0.73 ± 0.03, *p* = 0.0242; 0.51 ± 0.02, *p* = 0.0005; respectively, fold variation to control) compared to the cells of the control group (1.00 ± 0.15) (Figure 2a).

Regarding mitochondrial membrane potential, exposure to none of the liraglutide concentrations (25, 50, 100 nM) showed differences (0.94 ± 0.03, 0.92 ± 0.03, and 0.93 ± 0.02—fold variation to control, respectively) when compared to the control group (1.00 ± 0.02) (Figure 2b).

### 2.3. Pharmacological Concentration of Liraglutide Increases Basal Respiration, Proton Leak, Maximal Respiration, and ATP-Coupled Respiration of Mouse Leydig Cells

**Figure 3:**
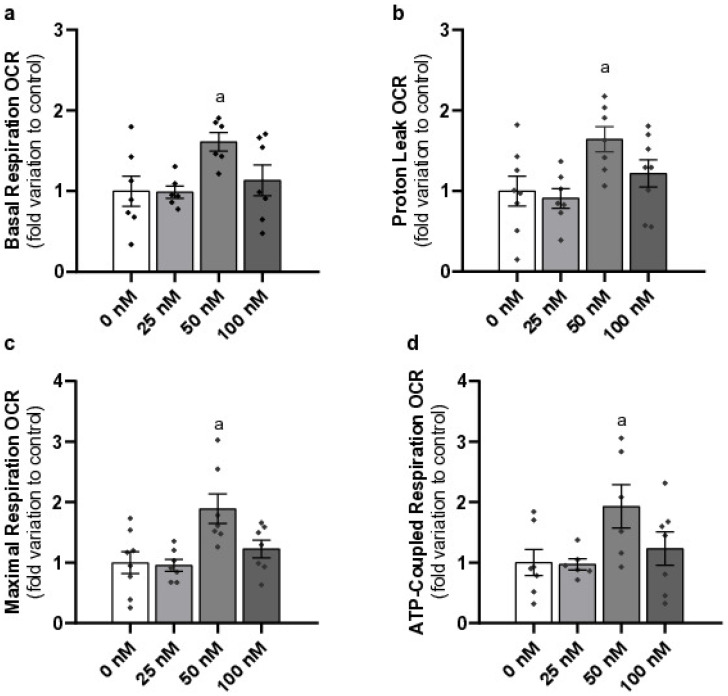
Effects of different concentrations of liraglutide on Leydig cell (LC) mitochondrial performance, Basal Respiration (**a**), Proton Leak (**b**), Maximal Respiration (**c**), ATP-coupled Respiration (**d**). LCs were exposed to sub-pharmacological (25 nM), pharmacological (50 nM), and supra-pharmacological concentrations (100 nM) of liraglutide. Results are expressed as mean ± SEM (*n* = 8 for each condition), in fold variation to the control group (0 nM liraglutide). Significantly different results (*p* < 0.05) are indicated as: a—relative to control (0 nM liraglutide), ^♦^—represent each *n*.

We further investigated the influence of liraglutide on LC mitochondrial performance by Seahorse XF Cell Mito Stress Test, which measures OCR in live cells and assesses several different mitochondrial parameters. Basal respiration was measured after exposure to different concentrations of liraglutide (25 nM–0.99 ± 0.08, 50 nM–1.61 ± 0.11, and 100 nM–1.12 ± 0.19—fold variation to control). Our results indicate that a pharmacological concentration of liraglutide significantly increased the basal OCR (*p* = 0.012) compared to the control group (1.00 ± 0.19) (Figure 3a). Similar results were observed in the proton leak in LCs treated with a pharmacological concentration of liraglutide (1.64 ± 0.16, *p* = 0.0097—fold variation to control) compared with the control group. No alterations were observed in the cells exposed to the sub- and supra-pharmacological concentrations of liraglutide (0.91 ± 0.12, 1.22 ± 0.17—fold variation to control, respectively) when compared to those from the control group (1.00 ± 0.18) (Figure 3b).

Maximal respiration OCR was also measured to determine the capacity of the mitochondria under stress conditions. We found a significant increase in maximal respiration OCR at 50 nM liraglutide (1.89 ± 0.24, *p* = 0.0014—fold variation to control) compared to the control (1.00 ± 0.18), indicating an enhancement in mitochondrial respiratory capacity. This parameter was not altered in the remaining experimental groups tested (25 nM–0.96 ± 0.10, 100 nM–1.23 ± 0.15—fold variation to control) (Figure 3c).

LCs treated with a pharmacological concentration of liraglutide (1.93 ± 0.36, *p* = 0.0172—fold variation to control) presented an increase in the OCR related to ATP-coupled respiration when compared to the cells from the control group (1.00 ± 0.22). The LCs treated with sub and supra-pharmacological concentrations of liraglutide (0.97 ± 0.09 and 1.23 ± 0.27—fold variation to control, respectively) did not present alterations when compared to the cells of the control group (Figure 3d).

### 2.4. Liraglutide Does Not Affect Mitochondrial DNA Copy Number or Androstenedione Production in Mouse Leydig Cells

**Figure 4:**
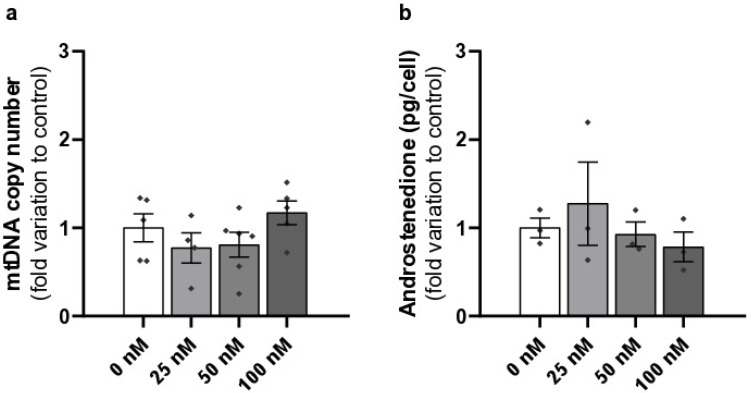
Effects of different concentrations of liraglutide on number of mtDNA copies (**a**) and androstenedione production (**b**) by Leydig cells (LCs). LCs were exposed to sub-pharmacological (25 nM), pharmacological (50 nM), and supra-pharmacological concentrations (100 nM) of liraglutide. Results are expressed as mean ± SEM (*n* = 5 for each condition), in fold variation to the control group (0 nM liraglutide). ^◆^—represent each *n*.

No significant changes were observed in the mitochondrial DNA copy number in cells treated with liraglutide at concentrations of 25 nM (0.77 ± 0.17—fold variation to control), 50 nM (0.81 ± 0.14—fold variation to control), and 100 nM (1.17 ± 0.14, fold variation to control) compared to those of the control group (1.00 ± 0.16). This indicates that liraglutide does not alter the replication or abundance of mitochondrial DNA in mouse LCs (Figure 4a).

Similar results were observed in androstenedione production by LCs exposed to different concentrations of liraglutide, indicating that liraglutide (25 nM–1.28 ± 0.47, 50 nM–0.93 ± 0.14, 100 nM–0.78 ± 0.17—fold variation to control) does not impair the production of androstenedione compared to the cells of the control group (1.00 ± 0.11) (Figure 4b).

## 3. Discussion

Over the past two decades, there has been a steady increase in the prevalence of metabolic diseases such as diabetes and obesity [22]. Given the growing global prevalence of these diseases, the pharmaceutical industry has developed various drugs to mitigate their effects. Several studies highlight the impact of these drugs on multiple systems, including the cardiovascular system [23] and the urinary system [24]. However, their impact on the reproductive system and fertility is often overlooked. The declining male fertility rates observed in recent years have been conclusively linked to the rising incidence of metabolic diseases [25]. Liraglutide is a GLP-1RA, widely used in the treatment of diabetic and obese patients, with significant improvements in both these clinical conditions [26,27]. GLP-1RAs not only improve metabolic control and promote weight loss, but have also been associated with increased total and free testosterone in men with functional hypogonadism. While part of this effect is attributable to weight reduction, accumulating evidence suggests a potential direct action on testicular function, as GLP-1Rs are expressed in testicular cells. In vitro studies show that GLP-1RAs improve sperm metabolism and motility, and may enhance Sertoli cell function, supporting a role in male reproductive health beyond weight loss alone [14,19,28].

However, there is a limited body of research that has shed light on the influence of this drug on male fertility, and the available studies are quite scarce. Indeed, to our knowledge, no studies focusing on the effect of liraglutide on LCs have been performed. Hence, in our study, we assessed the impact of liraglutide on LCs exposed to a normoglycemic environment, aiming to replicate in vitro the drug’s effects on individuals without diabetes, since patients with obesity and without diabetes also have this drug prescribed.

We observed that the metabolic viability of BLTK1 LCs increased three-fold when in the presence of pharmacological and supra-pharmacological concentrations of liraglutide compared to cells not exposed to liraglutide. One study had already described a similar result in β-cells, where liraglutide at a concentration of 100 nM significantly increased the metabolic viability of these cells [29]. Nevertheless, a separate study failed to replicate these findings in astrocytes, demonstrating that liraglutide at these concentrations had no discernible effect on their viability [30]. This can be correlated with the results obtained when evaluating mitochondrial fitness through Seahorse XF Mito Stress Tests, since metabolic viability is directly associated with mitochondrial function. After careful analysis of the results obtained in our study on the different mitochondrial parameters, we detected a pattern between basal respiration, maximal respiration, ATP-coupled respiration, and proton leak after exposure to the pharmacological concentration of liraglutide, since it increased all these parameters when compared to the control condition. Basal respiration is the level of oxygen consumed to meet the ATP demand of the cell after mitochondrial proton leak under baseline conditions. Its increase upon treatment with liraglutide at pharmacological concentration implies that LCs become more metabolically active and in need of more ATP for cellular physiology. The same applies to maximal respiration, which shows the maximum rate of respiration achievable by the cell and demonstrates how the cell manages to respond in a situation of high energy demand. In a study involving cardiomyocytes, it was also observed that treatment with the pharmacological concentration of liraglutide led to an increase in both basal and maximal respiration [31]. ATP-coupled respiration, calculated by the portion of OCR used for ATP production in the electron transport chain, which translates into more ATP being produced, was also increased in LCs subjected to the pharmacological concentration of liraglutide. It has been reported that mitochondrial ATP is vital for steroidogenic capacity and supports steroidogenesis. Allen et al. exposed MA-10 cells (an immortalized LC line) to oligomycin in growing concentrations to inhibit ATP synthesis and found that progesterone production and mitochondrial steroidogenic acute regulatory protein (StAR) expression were both reduced in a dose-dependent manner [32]. However, in the present study, no differences in androstenedione production by LCs subjected to liraglutide were observed. We propose that the increase in ATP production was insufficient to significantly alter the synthesis of steroids further downstream in the steroidogenesis cascade. However, it may have provided an energetic boost to LCs.

With regards to another extremely important mitochondrial parameter, proton leak, this represents the portion not directly linked to ATP production, in the context of basal respiration. While it is commonly associated with mitochondrial damage, it is important to note that this is not always the case; proton leak can also play a role in indirectly regulating ATP production [33]. Our results show that liraglutide has no cytotoxic effect in LCs and stimulates the metabolic activity of these cells. Additionally, we have also evaluated the production of ROS in LCs, which has been widely reported to induce proton leak [34]. However, we noticed a considerable decrease in ROS production in LCs after exposure to all liraglutide concentrations. Since the proton leak was increased, we can conclude that the cause for that was not the abundance of ROS. Like other authors [35], we propose the existence of a protective feedback loop in which ROS might increase proton leak, and increased proton leak in turn lowers ROS formation to prevent more harm to mitochondrial function. Nevertheless, decreased ROS production upon treatment with liraglutide has been broadly reported in other cell types. Chen et al. [36] described that an OS-induced cardiomyoblast cell line had decreased ROS production after pretreatment with liraglutide at 100 nM through an augmented mRNA abundance and protein expression of sirtuin 1, known as a powerful attenuator of NADPH oxidase 4, which is involved in ROS generation [37].

Although some studies link increased mitochondrial bioenergetics with mtDNA copies [38], we observed that liraglutide does not affect mtDNA copies in LCs. We can conclude that the pharmacological concentration of liraglutide improves mitochondrial bioenergetics but is not related to the number of mtDNA copies. As for the analysis of steroidogenesis, we did not evaluate testosterone production in these cells because, according to Engeli et al. [39], the mRNA of the enzyme needed in the final step of steroidogenesis for conversion of androstenedione into testosterone, 17β HSD3, is extremely poorly expressed in LCs, in comparison to mouse testis, which expresses it 1000 times more. Moreover, none of the currently available mouse LC lines sufficiently express this enzyme to produce testosterone in high enough quantities to be detected by an ELISA assay [39]. In this study, we observed that androstenedione production by LCs is not affected by liraglutide, but this result might be owed to cell line limitations (basal 17β HSD3 expression). Proper conclusions regarding the effect of liraglutide in the testosterone synthesis of LCs require validation in primary LCs.

Hence, it becomes evident that LCs cultivated in vitro and subjected to a pharmacological concentration of liraglutide exhibit heightened metabolic activity, as evidenced by the amplified mitochondrial function in addition to reduced ROS production. These enhancements could potentially serve as a favorable attribute contributing to the optimal function of mouse LCs. Furthermore, extrapolating these results to humans, liraglutide at the pharmacological concentration seems to have a beneficial effect on the LCs of patients, making it a safe antidiabetic and anti-obesogenic that does not disrupt male fertility in this context. Overall, these findings reinforce the potential of GLP-1RAs, in particular liraglutide, not only as metabolic therapies but also as agents with favorable implications for male reproductive health, offering a dual benefit that conventional testosterone replacement cannot provide.

However, some study limitations should be acknowledged. Importantly, we did not identify GLP-1R expression in BLTK1 LCs, despite it being present in other LC lines, such as TM3 [13]. Nevertheless, liraglutide exhibits pleiotropic effects [40] and may exert its action through non-canonical pathways [41], namely by interaction with caveolin-1 [42], highlighting the complexity of the role of GLP-1 metabolic regulation, and suggesting that its actions are not solely dependent on direct GLP-1R activation. However, we observed significant effects with the use of liraglutide, which suggests that its action may occur either through the GLP-1R pathway or through an unconventional pathway. In addition, as mentioned above, the limited LC steroidogenesis in immortalized mouse LC lines owing to the basal expression of 17β HSD3 [39] propelled us to measure androstenedione instead of testosterone. Furthermore, we did not directly assess ATP or ADP/ATP quantity, although ATP-coupled respiration was significantly altered upon treatment with a pharmacological dose of liraglutide. Even though we detected a decrease in ROS production, we did not identify which specific ROS were affected. Finally, it is important to note that further validation of these results in an in vivo setting in mice and primary and human-derived models is imperative.

## 4. Materials and Methods

### 4.1. Cell Culture and Experimental Groups

LCs line (BLTK1 cell line) was obtained from a murine testicular tumor that developed in a transgenic mouse expressing the mouse inhibin-α promoter/simian virus 40 T-antigen fusion gene [43]. The cell line was kindly provided by Prof. Nafis Rahman and Prof. Ilpo Huhtaniemi. The BLTK1 cell line has been widely used in in vitro studies; however, like other available Leydig-derived cell lines, it is not an ideal model. Similar to MA-10 and TM3 cells, BLTK1 cells exhibit impaired androgen biosynthesis, characterized by negligible testosterone production [39]. Briefly, cells were seeded in 75 cm^3^ flasks in a 1:1 mixture of Ham’s F12 medium and Dulbecco’s modified Eagle’s medium (DMEM) with 3.57 g/L HEPES, 1.2 g/L sodium bicarbonate, 50 U/mL penicillin, 50 U/mL streptomycin sulfate, 50 µg/mL gentamicin, and 10% fetal bovine serum (FBS). We also added 3 ng/mL human chorionic gonadotropin (hCG) to all groups to induce testosterone production (mimics LH) [23]. Cells were grown until they reached 70–80% confluence at 37 °C in an atmosphere of 5% CO_2_:95% O_2_.

To study the effect of liraglutide (24727, Cayman Chemical, Ann Arbor, MI, USA) in LCs, we designed 4 experimental groups. Cells were treated without (control group) or with sub-pharmacologic, pharmacologic, or supra-pharmacologic concentrations of liraglutide (25 nM, 50 nM, and 100 nM, respectively) [44,45] in normoglycemic conditions (glucose 5 mM) for 48 h. A vehicle control containing 0.1% ethanol (vehicle used for liraglutide) was included as a control group, as this concentration is widely used as a standard solvent condition and is considered non-toxic to cells, ensuring that any observed effects are attributable to the test compounds rather than the vehicle itself.

### 4.2. Viability Assays

#### 4.2.1. Sulforhodamine B (SRB) Cytotoxicity Assay

BLTK1 LCs proliferation upon liraglutide exposure during 48 h was evaluated by SRB cytotoxicity assay. Cells were seeded in 24-well plates until they achieved 70–80% confluence. After the 48-h incubation with the different concentrations of liraglutide, the cells were fixed with a 1% acetic acid in 99% methanol solution for 1 h at −20 °C. Afterwards, cells were incubated with a 0.05% SRB solution with 1% acetic acid in water for 1 h at 37 °C. After a washing step, the SRB dye was removed from the cells with a 10 mM Tris solution with pH 10, and the optical density was determined at 490 nM using the Biotek Synergy H1 Microplate Reader (BioTek, Winooski, VT, USA) [46]. The resulting values of each treated group were presented as fold variation to the control group.

#### 4.2.2. Lactate Dehydrogenase (LDH) Release Assay

Cells were plated in 24-well plates and exposed to the different liraglutide concentrations to assess extracellular lactate dehydrogenase (LDH) release. LDH release was determined by measuring the extracellular activity using a commercial assay kit following the manufacturer’s instructions (LDH CitoxTM Assay Kit, BioLegend^®^, San Diego, CA, USA). After 48 h of treatment, 100 µL of culture medium from each well was placed into a 96-well plate and incubated with 100 µL LDH working solution for 30 min at 37 °C away from light. A 50 µL stop solution was then added to stop the enzymatic activity, and absorbance was measured at 490 nM using the Biotek Synergy H1 Microplate Reader (BioTek, Winooski, VT, USA). LDH release of each treated group was presented as fold variation to the control group.

#### 4.2.3. MTT Viability Assay

BLTK1 LC viability was assessed by MTT (3-(4,5-dimethylthiazol-2-yl)-2,5-diphenyl-2H-tetrazolium bromide) assay. Cells were cultured in 48-well plates and left to grow until they reached 70–80% confluence. After 48 h of incubation with the different concentrations of liraglutide, the medium was replaced with 500 µL growth medium and 50 µL MTT solution (5 mg/mL) and incubated for 2 h at 37 °C protected from light. The medium was then removed, and the MTT crystals dissolved in 250 µL of dimethyl sulfoxide (DMSO). The absorbance was measured at 570 nM and 655 nM in the Biotek Synergy H1 Microplate Reader (BioTek, Winooski, VT, USA). Metabolic viability was calculated by the difference between 570 nM and 650 nM. After this adjustment, the blank was removed in each sample (well with only DMSO), and the results are expressed as fold variation to the control group.

### 4.3. General Oxidative Stress Indicator Assay

Intracellular ROS levels in BLTK1 LCs were measured using CM-H2DCFDA (C6827, Thermo Fisher, Waltham, MA, USA). Cells were cultured until they reached 70–80% confluence in 96-well black plates and then treated with different concentrations of liraglutide for 48 h. We then prepared the CM-H2DCFDA at 5 µM probe and HOECHST (1 µg/µL) (33342, Thermo Fisher, USA) to stain nuclei cells. With the probe fully prepared for use, the cells were incubated with it for 30 min at 37 °C away from light. Finally, the fluorescence was read at Ex 495 nM/Em 529 nM (red) and Ex 350 nM/Em 461 nM (blue). Results were expressed as the ratio of red/blue and expressed in fold variation to the control group.

### 4.4. Gene Expression

#### 4.4.1. DNA Extraction

Total DNA was extracted from BLTK1 LCs after treatment with different concentrations of liraglutide, using the QIAmp DNA Mini Kit according to the manufacturer’s instructions. Total DNA was then quantified using a BioTek^®^ SYNERGYH1 microplate reader (Bio-Tek^®^, Agilent Instruments, Winooski, VT, USA) with the Gen5TM Data Analysis Software 2.0 (BioTek^®^, Agilent Instruments, USA).

#### 4.4.2. Quantitative Real-Time PCR (qPCR)

Quantitative Real-Time PCR (qPCR) was performed to evaluate the number of mtDNA copies in the cells of all the groups previously described through the abundance quantification of the mitochondrial gene *MT-ND1*. Specific primers were designed for the amplification of the β-2-microglobulin (housekeeping gene) and *MT-ND1* genes, as described in Table 1. qPCR conditions were previously optimized, and the specificity of the primers was determined by melting curves. Amplification conditions were 5 min at 95 °C, followed by 30–35 runs of a three-step cycle: 10 s at 95 °C, 30 s with a specific temperature for each set of primers, and 10 s at 72 °C. β-2-microglobulin gene levels were used to normalize *MT-ND1* gene abundance levels. Fold variation of gene abundance levels was calculated following the model proposed by Pfaffl [47].

### 4.5. Mitochondrial Membrane Potential Assay

BLTK1 LCs mitochondrial membrane potential was evaluated by the dye 5,5′,6,6′-tetrachloro-1,1′,3,3′-tetraethylbenzimidazolylcarbocyanine iodide (JC-1). Cells were seeded and allowed to grow in black 96-well plates until 70–80% confluence. After 48 h of incubation with the different concentrations of liraglutide, the medium was discarded and substituted with growth medium containing the fluorescent probe JC-1 at 2 µM for 30 min at 37 °C away from any light sources. JC-1 monomers (485/530 nM; Ex/Em) and J-aggregates (535/390 nM; Ex/Em) fluorescence were read on a Synergy™ H1 multi-mode microplate reader (BioTek, Winooski, VT, USA). Results were expressed as the ratio of aggregates/monomers, and presented in fold variation to the control group.

### 4.6. Cellular Oxygen Consumption Analysis

The oxygen consumption rate (OCR) of BLTK1 cells was measured by the Agilent Seahorse XFe24 Analyzer (Agilent Technologies, Inc., Santa Clara, CA, USA) using the Agilent Seahorse XF Cell Mito Stress Test Kit (Agilent Technologies, Inc., Santa Clara, CA, USA). BLTK1 cells were cultured in Seahorse XF24 cell culture microplates and allowed to grow until 70–80% confluence. The growth medium was then substituted with different treatment mediums for 48 h. For standard mitochondrial oxygen consumption assessment, the following compounds were used: oligomycin (1.5 µM), fluorocarbonyl cyanide phenylhydrazone (FCCP, 1 µM), and rotenone/antimycin A (0.5 µM). We set up the following protocol to measure BLTK1 OCR: Equilibration (short mixing period—3 min), where the first set of measurements were recorded under basal conditions; Injection 1 (oligomycin), 2 (FCCP), and 3 (rotenone/antimycin A) with 3 cycles, with 30 s of mixing, 2 min of wait, and 3 min of measurement per cycle. Between each injection, three measurements of each OCR were recorded. OCR data were normalized by total protein content. Results were expressed in fold variation to the control group.

### 4.7. Enzyme-Linked Immunosorbent Assay (ELISA)

Androstenedione production by BLTK1 cells was assessed by ELISA kit (ab108672, Abcam, Cambridge, UK) according to the manufacturer’s instructions. Briefly, medium was collected from 6-well plates after 48 h of treatment, and 25 µL was placed in each well of the assay plate, followed by 200 µL of the androstenedione-HRP conjugate. The plate was then incubated for 1 h at 37 °C away from light. The wells were then washed, and 100 µL of TMB substrate was added to each well. Finally, after the addition of 100 µL of stop solution, the absorbance was read at 450 nM. The absorbance readings were converted into androstenedione concentration (ng/µL) using Four Parameter Logistic. Results were expressed in fold variation to the control group.

### 4.8. Statistical Analysis

Statistical significance between the samples from the different experimental groups was assessed by using an ordinary one-way ANOVA in addition to uncorrected Fisher’s LSD, with a single pooled variance test. Data from all experiments are presented as mean ± standard error of the mean (SEM). Statistical analysis was performed using GraphPad Prism 8 (GraphPad Software, San Diego, CA, USA). Outliers were identified using the method of Grubbs with alpha equal to 0.2. Results were considered significant when *p* < 0.05.

## Figures and Tables

**Table 1:** Oligonucleotides and cycling conditions for PCR amplification of *ND1* and β-2-microglobulin (B-2M) genes.

Gene	Sequence 5′-3′	Annealing T°	Cycles
*ND1*	FWD: CATCTTATCCACGCTTCCG RVS: GTGGTACTCCCGCTGTAA	60 °C	35
*β-2-M*	FWD: GTAACACAGTTCCACCCG RVS: TCGATCCCAGTAGACGGT	58 °C	30

## Data Availability

The data presented in this study are available on request from the corresponding authors.
